# Combining and Using the Utrecht Method and the Analytic Hierarchy Process to Facilitate Professional and Ethical Deliberation and Decision Making in Complementary and Alternative Medicine: A Case Study among a Panel of Stakeholders

**DOI:** 10.1155/2018/2315938

**Published:** 2018-12-23

**Authors:** Ramzi Shawahna

**Affiliations:** ^1^Department of Physiology, Pharmacology and Toxicology, Faculty of Medicine and Health Sciences, An-Najah National University, Nablus, State of Palestine; ^2^An-Najah BioSciences Unit, Centre for Poisons Control, Chemical and Biological Analyses, An-Najah National University, Nablus, State of Palestine

## Abstract

**Background:**

In daily practice, healthcare practitioners face many challenges in ethical and professional decision making. Currently, little is known on the ethical and professional deliberations and weighing benefits against risks in daily complementary and alternative medicine (CAM) practice. The aim of this study was to combine the Utrecht method and the Analytic Hierarchy Process (AHP) in deliberations, weighing benefits against risks of using ginger for a pregnant woman suffering nausea and vomiting of pregnancy (NVP) along with other comorbidities.

**Methods:**

A hypothetical case was constructed using the twelve tips for constructing dilemma case-based assessment. Three CAM practitioners, two physicians, three pharmacists, and two patients were recruited, and the Utrecht and the AHP methods were combined and used to deliberate and weigh benefits against risks of using ginger for the presented case.

**Results:**

Responses from the ten panelists were obtained. Priority ratings showed significantly higher scores (*p*-value < 0.001) for alleviating symptoms of NVP (30.7%  ± 16.6%) compared to other potential benefits. Increasing the risk of bleeding was given significantly higher (*p*-value < 0.0001) weight scores (24.7%  ± 13.5%) than other potential side effects. Potential risk of spontaneous abortion and risk of impairment of fetal development were given higher (*p*-value < 0.001) weight scores than risk of fetal hypoglycemia. When benefits were compared against side effects and risks to the fetus and pregnancy, potential benefits were given higher (*p*-value < 0.001) weight scores (72.3%  ± 5.2%).

**Conclusions:**

Considering the anticipated benefits and risks, a shared decision was made to use ginger in the case presented. The woman should also be informed of the potential side effects and risks of using ginger. The use of this combined method might promote openness and transparency in making shared decisions for healthcare providers and patients.

## 1. Introduction

Nausea and vomiting of pregnancy (NVP) is one of the most prevalent complains of pregnant women during the first trimester of pregnancy [1]. It has been estimated that about 90% of pregnant women are affected by NVP to some degree [2]. The intensity of the symptoms often peak at 7–12 weeks and may subside by week 16. Symptoms persist beyond week 20 in about one-third of all pregnancies [1, 3]. In about 2% of pregnancies, women might develop* hyperemesis gravidarum* which is the most severe form of NVP [1–3]. If left untreated, this condition has serious consequences on the woman and her developing fetus [4–6]. These consequences include dehydration, electrolytes imbalance, damage to the liver, and fetal developmental abnormalities and in some cases might lead to the death of the woman and/or her fetus.

There are many cases in daily clinical practice in which pregnant women and healthcare providers are reluctant to use conventional medications, particularly, in the first trimester of pregnancy [3]. In these cases, women and/or their healthcare providers might opt for some modalities of complementary or alternative medicine (CAM), for example, using ginger to treat NVP [7]. Medicinal plants have evolved as one of the most frequently used CAM modalities. In many cases, medicinal plants are generally regarded as safe [7]. Probably, this notion stemmed from advertising medicinal plants as safe and gentle [8]. Moreover, some healthcare providers helped perpetuate this myth when recommending medicinal plants as “natural” remedies that are always safer than conventional medicines [9, 10]. Contrary to the beliefs of many patients, medicinal plants contain many chemical constituents that can be identical to those present in conventional medicines. In such case, these constituents may act in the same pharmacological mechanisms and hence have similar potential to cause unwanted side effects in a similar way to other conventional medicines. Therefore, like conventional medicines, medicinal plants should be given for certain indications, are contraindicated and should be used with caution in many cases, and might cause unwanted side effects. Consequently, medicinal plants should be recommended for the right patients, at the right time, in the right dose, at the right frequency, and by the right route of administration [7, 9].

Making decisions on certain therapeutic options when alternatives are available requires weighing their potential benefits against their potential risks. The therapeutic option is often chosen when its potential benefits outweigh its potential risks. Today, terms like choice, self-management, concordance, and informed decision making are key words being utilized in managing different health related conditions [11, 12]. Today, the patient reported outcomes are of utmost importance because improving the quality of life of the patient is the ultimate goal of any treatment option. Recognizing these principles changed the philosophy and practice of healthcare delivery. Consequently, patients are increasingly involved in weighing potential benefits against potential risks of the available treatment modalities, and shared decisions are more frequently made by patients and their healthcare providers.

In daily clinical practice, making decisions on therapeutic alternatives can be complicated, especially, in the presence of other comorbidities. While some patients might delegate the decision making process to their healthcare providers, many of them would prefer a more collaborative approach to make shared decisions. In both cases, the patient needs to be informed with the process of weighing benefits against risks of a treatment option. It can be argued that well-informed patients might have less misconceptions about the treatment and the anticipated outcomes, recognize the potential benefits, cope better with the unwanted side effects, and feel in control of their own lives [11, 13–17]. CAM is no exception and healthcare providers frequently face challenges to decision making in daily practice. Although the ethical aspects of professional practice are either explicitly mentioned or at least assumed, professional guidelines do not offer tools for professional and ethical deliberations and weighing potential benefits against potential risks in daily practice [18].

In general, the literature narrates little on ethical and professional deliberations in daily practice when opting for either conventional or CAM treatment options. The Dutch Centre for Bioethics and Health Law has developed a method known as Utrecht method which can be used in ethical and professional deliberations [19–21]. This method is frequently used in education. Given the complexity of weighing potential benefits against potential risks and making decisions, the use of a combination of qualitative and quantitative approaches that integrate potential benefits and risks and enable ranking certain benefits and/or risks should be useful to support decision making [22]. The literature reported on different multicriteria decision analysis (MCDA) approaches to support decisions in the face of uncertainties, especially, when many objectives related to the outcomes of a certain treatment were available [23]. Among these approaches, the Analytic Hierarchy Process (AHP) has emerged as one of the most commonly used. The AHP provides a means of explicitly incorporating benefits and risks of a treatment and combines the importance of differences in priorities of the treatment outcomes [24]. Contrary to the standard decision making processes in which the importance of each component of the decision is not explicit, the AHP allows a transparent decision making process in which the stakeholders can understand and demonstrate the bases of their decisions [24].

Little was reported on the use of deliberations and MCDA techniques to support decisions on CAM use. This study reports on combining and using the Utrecht method as a deliberation method and the AHP to support a decision to whether recommend ginger or not for a pregnant woman suffering NVP along with other comorbidities.

## 2. Methods

In this study, a combination of the Utrecht method and AHP was used to generate and analyze the views of a panel of pharmacists, physicians, and pregnant women on a hypothetical case of a pregnant women suffering NVP to decide whether to recommend ginger for her or not after prioritizing potential benefits and risks to herself, her pregnancy, and her developing fetus. This case was developed considering the twelve tips for constructing a dilemma case-based assessment [25]. These tips were* (1) selecting an appropriate practice/ethical issue, (2) using a case with “true” dilemma, (3) targeting high-level cognitive tasks, (4) developing a list of key components, (5) providing a single central theme, (6) devising a scoring system that is understandable to the stakeholders, (7) the case dilemma and modifier factors being important and plausible, (8) being clear, (9) selecting qualified stakeholders, (10) conducting validation and piloting, (11) being aware of the limitations, and finally (12) the developer having sufficient knowledge and understanding of the concepts being tested*.

### 2.1. The Case

A 37-year-old pregnant women in her 7^th^ week of gestation presents with NVP. This was not her first pregnancy; she has three healthy children. Her previous pregnancies were a nightmare as a result of NVP. She is reluctant to take prescription medications for her NVP and prefers some “natural” remedy. Her friends told her that ginger (*Zingiber officinale* Roscoe) might help subside her symptoms. Recently, she caught flu and now she coughs and has nasal congestion. Her medical history shows that she has type II diabetes mellitus and a mild hypertension. She is on antihypertensive medications, oral hypoglycemic agents, insulin, a statin, and a low dose aspirin. Although she tried to breastfeed her three children, in every time her milk was not enough to totally depend on and feeding was supplemented with formula. The woman is overweight and often suffers joints pain. Her previous attempts to lose weight were futile as she could not control her appetite. Although she was not screened for ulcer, she often suffers heartburns and dyspepsia, especially with certain foods. She works as an accountant and often complained having difficulty falling asleep. She always wanted to seek a medical advice for this problem but did not have the opportunity to see a physician for this problem.

### 2.2. The Utrecht Method

This method was originally developed as a reflective tool for deliberation with special focus on professional and/or ethical dilemmas facing healthcare providers in daily practice [21]. Deliberations are often started with action-guiding questions such as “*what I am supposed to do?*” that finally lead to concrete advice. Decisions are justified through reasons sought as professional or ethical decision making that requires transparency. This method takes into account the different normative viewpoints that healthcare professionals as well as patients might hold in practice which could be considered during the deliberation process. The use of the Utrecht method could be attractive as it fits with daily professional practice and the limited amount of questions posed during the deliberation process. There are eight questions in this method, which were as follows:* first, what is the professional and/or ethical question in this case? Second, what are the alternative potential decisions in this case? Third, is there a lack of relevant information? Or what are the potential benefits and potential risks of using ginger in this case? Fourth, what are the perspectives of the stakeholders on this case? Fifth, what are the arguments for and against the alternative potential decisions? Sixth, how strong are these arguments in this case? Seventh, which decision alternative is preferred based on the arguments considered in this case?* And finally* eighth, how to implement the decision preferred for this case*? Deliberation questions 1-6 are presented in the Methods section below, question 7 is presented in the Results section, and question 8 is presented in the Conclusion section.

#### 2.2.1. What Is the Professional and/or Ethical Question in This Case?

Although this case might lead to multiple professional and ethical questions, the question suggested was “*should this woman use ginger?*” This question was posed in neutral and concrete manner and it does not contain any professional or ethical arguments.

#### 2.2.2. What Are the Potential Alternative Decisions in This Case?

The potential alternative decisions in this case are identified without any final decisions made. These potential alternative decisions could be as follows:* (1) this woman is recommended to use ginger without any further discussions, (2) this woman is recommended to use ginger after verifying that she is well-informed and understands the risks of using ginger in her case, (3) making a shared decision between this woman and the healthcare provider to use ginger when considering the risks are negligible, or (4) making a shared decision to avoid using ginger in her case. *

Obviously, there were differences in priorities for each alternative decision that need to be considered and which were not immediately clear to make a decision at this stage.

#### 2.2.3. Is There a Lack of Relevant Information? Or What Are the Potential Benefits and Potential Risks of Using Ginger in This Case?

Professional as well as ethical decisions should be substantiated with relevant information. Therefore, all relevant information regarding the potential benefits and risks of using ginger in this case should be understood thoroughly before making any decision. In a previous study, a Delphi technique was followed among gynecologists, other physicians who frequently see pregnant women with NVP, and pregnant women to achieve consensus on the potential benefits and risks of using ginger for NVP that should be addressed in a clinical consultation [7]. The potential benefits and harms were obtained from interviews with gynecologists, physicians, and women as well as from the literature [1–3, 9, 26–47].

It was important to take into consideration that the efficacy and safety of ginger in managing NVP are still indecisive considering a number of conflicting reports [1, 31]. Previous studies showed that ginger decreased aggregation of platelets, increased acid production in the stomach, and had the potential to interact with many medications [1, 29, 37, 48]. A previous study demonstrated that ginger contains gingerols that have the ability to inhibit arachidonic acid-induced human platelet serotonin release and aggregation in a potency comparable to that of aspirin [36]. The compound 8-paradol, which is one of the constituents of ginger, has a relatively potent inhibitory action on the cyclooxygenase (COX-1) and has antiplatelet aggregation activity [38]. Bleeding, especially in the first trimester could be severely detrimental to pregnant women and their fetuses and in many cases was shown to lead to miscarriage [49]. Although ginger is often labeled as “generally regarded as safe” in the US, in Germany and Finland, some health regulatory agencies have advised against using ginger in pregnancy [9, 26].

Some studies alluded to a link between ginger and increased risk of spontaneous abortion and fetal developmental abnormalities [30, 32, 33, 41]. These suggestions are contradictory to what Portnoi et al. showed in Canada, that the use of ginger in the first trimester did not increase the risk of spontaneous and therapeutic abortions in 187 women exposed to ginger compared to 187 women who were not exposed to ginger [40], and those shown in a larger study conducted in Norway [34]; however, in conservative views, these results should be considered with caution and patients as well as healthcare providers might take into consideration an inconclusive link between using ginger and risk on the fetus and continuity of the pregnancy [7]. Ginger was also shown to reduce blood pressure and blood sugar levels [27, 28, 39, 43]. However, the effects of ginger on blood sugar are expected at higher doses than those recommended for NVP and might take months to appear [50]. A previous study showed that ginger extracts blocked voltage-dependent calcium channels and lowered blood pressure in animal models [51]. These findings in laboratory animals were contradictory to those shown in a randomized clinical trial in which ginger, among other herbal preparations, did not bring about significant changes to blood pressure of patients with type II diabetes mellitus [52]. Therefore, it is not clear if the use of ginger might potentiate the effects of insulin and antihypertensive medications and might necessitate adjustment of the doses.

Ginger was also shown to be associated with cardiac arrhythmias, sweating, thirst, dehydration, duodenal ulcer, diarrhea, mild headache, fever, mild skin itching, belching, and heartburns [1, 27, 28, 39, 43]. On the other hand, ginger might help alleviate cough, flu, joints pain, and dyspepsia. Ginger might also induce somnolence, help reduce hypercholesterolemia, increase milk production, improve skin, help controlling appetite, and promote weight loss.

#### 2.2.4. What Are the Perspectives of the Stakeholders on This Case?

The stakeholders in this case were the woman herself and her caring healthcare provider. This healthcare provider could be a physician, pharmacist, herbalist, or another healthcare providers. For this study, a panel of 10 participants was composed. The panelists were 2 physicians, 3 pharmacists, 3 herbalists, and 2 pregnant women. The panelists were provided with the case as well as the potential benefits, side effects, and risks of using ginger in this case. Perspectives of the panelists on the potential benefits, side effects, and risks when appraising benefits and avoiding side effects and risks were obtained.

#### 2.2.5. What Are the Arguments for and against the Alternative Potential Decisions?

For this case, there were multiple relevant potential benefits and harms of ginger. Ginger has anticoagulant activity, although at first this appears to be beneficial for her case, she was already taking a low dose aspirin as an antiplatelet agent. Therefore, ginger might potentiate the anticoagulant activity of aspirin and might increase the risk of bleeding. This increased risk should be taken into consideration. Previous studies alluded to a link between ginger and increased risk of spontaneous abortion and fetal developmental abnormalities [30, 32, 33, 41]. However, it is important to take into consideration that these allegations were contradictory to findings of larger studies in Canada and Norway [34, 40]. In the presented case, the woman had three healthy children. This woman was not formally diagnosed with duodenal ulcer, but she often suffers heartburns and dyspepsia. Ginger has the potential to worsen duodenal ulcer and heartburns. However, it can be beneficial for dyspepsia which this woman suffers from. The medical history of this woman shows that she is diabetic and hypertensive. She is on insulin, statin, antihypertensive, and oral hypoglycemic medications. The effects of ginger on blood pressure, cholesterol, and sugar levels are not conclusive given the recommended dose of ginger for NVP. It is not clear if ginger might reverse these conditions already controlled with medications. Ginger might also be associated with other side effects like dehydration, thirst, fever, sweating, skin itching, and belching. However, these side effects are not commonly associated with doses of ginger used for NVP [50]. On the other hand, ginger might be beneficial in alleviating the symptoms of her NVP, cough, flu, and joints pain. Ginger can also be beneficial in increasing her milk production, control her appetite and help her lose weight, and improve her sleep.

#### 2.2.6. How Strong Are These Arguments in This Case?

To investigate how strong these arguments are in this case, the AHP was used. In AHP, panelists use pairwise comparisons to weigh alternatives and facilitate decision making. In this study, the goal was to determine relative weights of benefits to appraise therapeutic effects and avoid side effects and risks on the continuity of pregnancy and the integrity of the developing fetus. The panelists were provided with summaries containing information on safety and efficacy of ginger in NVP. Data relevant to the safety and efficacy of ginger were summarized from Natural Medicines Comprehensive Database [50], Cochrane Database of Systematic Reviews [53], and summary of relevant systematic reviews and research papers [1, 27–34, 36–41, 43, 48]. The panelists were provided with full text copies of the papers whenever they requested them. The panelists were requested to make pairwise comparisons on a scale of 9-points. The higher the numerical value given to an item (benefit, side effect, or risk), the higher the relative weight of the item compared to the other item being compared with. The panelists were requested to consider the probability of each benefit, side effect, or risk relevant to the case presented while making the pairwise comparisons. The comparisons were conducted in 4 stages. In the first stage, the panelists were requested to rate the weights of the potential benefits. In this stage, 12 potential benefits were compared in pairwise. These benefits were alleviating NVP, alleviating cough, alleviating flu, increasing milk production, decreasing appetite, decreasing cholesterol levels, reducing blood pressure, reducing blood sugar levels, alleviating dyspepsia, improving sleep, improving skin health, and reducing joint pain. In the second stage, the panelists were requested to rate 15 potential side effects in pairwise. These side effects were risk of bleeding, cardiac arrhythmia, irritable bowel syndrome, duodenal ulcer, heartburns, hypotension, hypoglycemia, skin itching, dehydration, belching, thirst, sweating, fever, headache, and diarrhea. In the third stage, the panelists rated 3 potential risks on the continuity of the pregnancy and the integrity of the developing fetus in pairwise [1, 27–34, 36–41, 43, 48]. These risks were risk of spontaneous abortion, risk of impairment of fetal development, and risk of fetal hypoglycemia. In the last stage, potential benefits, side effects, and risks were compared in pairwise comparisons. Individual ratings from each panelist were used to compute the comparison matrices in Excel spreadsheets [54]. Relative weight scores as well as their consistency ratios were calculated using the mathematical formulas originally developed by Saaty [55].

### 2.3. Analysis of Data

When the consistency ratios of the ratings were > 0.1, they were excluded from the final analysis. Data were entered in GraphPad Prism 6.0 for Windows (GraphPad Software). To assess overall differences in ratings, one-way analysis of variance (ANOVA) with Bonferroni post hoc tests was used to compare the ratings. The statistical significance was considered when the* p*-value was < 0.05.

### 2.4. Ethical Approval

The present study received approval of the Institutional Review Board (IRB) of An-Najah National University. The ten panelists gave verbal consent before they took part in this study.

## 3. Results

Ratings were obtained from the 10 panelists (response rate = 100%). The two physicians were females and gynecologists. Both had practicing experience of more than 10 years and often see pregnant women and counsel them on the safe use of medicinal plants including ginger for NVP. The three pharmacists were two females and one male. They also had practicing experience of more than 10 years in community pharmacy settings. They often dispense medications to pregnant women and often discuss with pregnant women the safety issues of medicinal plants. The three herbalists were all males with more than 15 years of practicing experience. They often sell medicinal plants to pregnant women and instruct them how to prepare them. The two pregnant women had more than 3 previous pregnancies. One of them had a history of miscarriage.

### 3.1. Which Decision Alternative(s) Is (Are) Preferred Based on the Arguments Considered in This Case?

**Figure 1:**
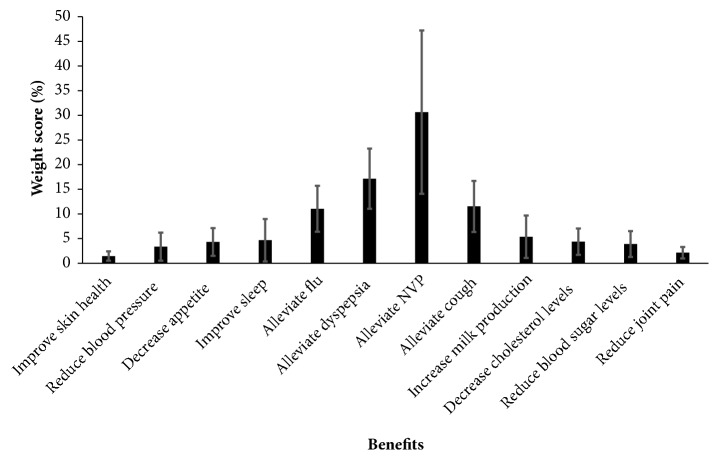
Weight scores of the benefits of ginger for the case presented.

Treatment prioritization analysis was performed on the grounds of analyzing benefits of ginger for this case, potential side effects, and risks to the fetus and pregnancy. Analyzing ratings on the benefits of ginger in this case showed that alleviating symptoms of NVP had the highest weight scores (30.7%  ± 16.6%) and the one-way ANOVA with Bonferroni post hoc multiple comparisons showed that this weight score was significantly higher than other weight scores (*p*-value < 0.001). Therefore, alleviating symptoms of NVP was ranked 1^st^. Multiple comparisons showed statistically significant difference in weight scores of alleviating symptoms of dyspepsia compared to all other benefits (*p*-value < 0.001) except for alleviate cough (*p*-value = 1.000) and flu (*p*-value = 0.890). Scores for alleviating symptoms of cough and flu were not significantly different (*p*-value > 0.05), while they were statistically different from reducing joint pain (*p*-value < 0.05) and improving skin health (*p*-value < 0.01). Detailed weight scores of the potential benefits are shown in Figure 1. Multiple comparisons are presented in Supplementary Table S1.

**Figure 2:**
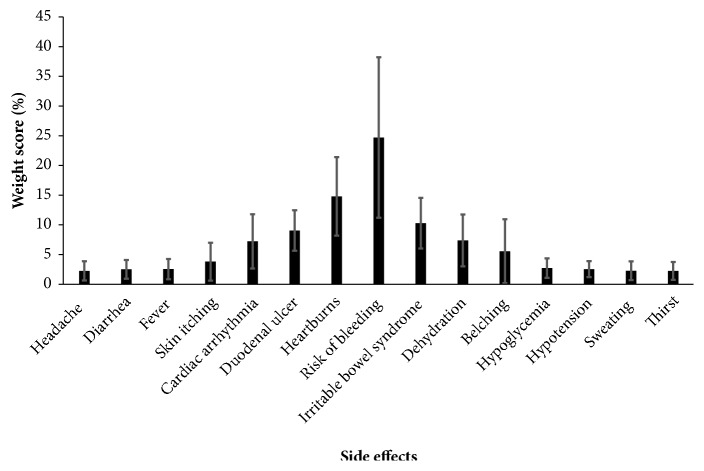
Weight scores of the side effects of ginger for the case presented.

Analyzing ratings of the side effects showed that the risk of bleeding was given significantly higher weight score (*p*-value < 0.001) than others (24.7%  ± 13.5%) and was ranked 1^st^. Scores of cardiac arrhythmia and dehydration were not statistically significant (*p*-value > 0.05). Scores of heartburns (14.8%  ± 6.6%) were significantly higher than all other potential side effects (*p*-value < 0.01) except duodenal ulcer (*p*-value = 0.145) and irritable bowel syndrome (*p*-value = 1.000). Detailed weight scores of the potential unwanted side effects are shown in Figure 2. Multiple comparisons are presented in Supplementary Table S1.

**Figure 3:**
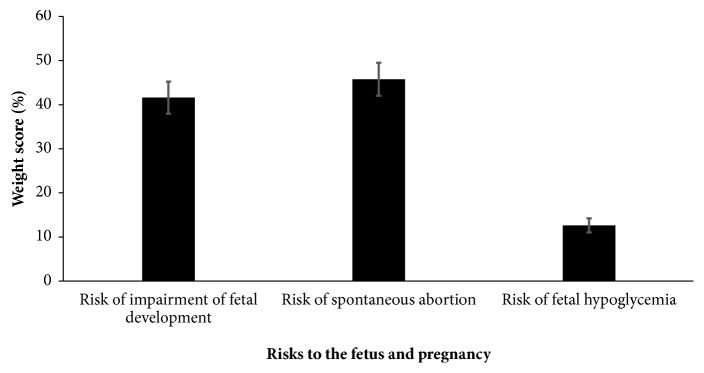
Weight scores of the risks of ginger to the fetus and pregnancy in the case presented.

Weight scores for risk of spontaneous abortion (45.8%  ± 3.8%) and risk of impairment of fetal development (41.6%  ± 3.6%) were significantly higher (*p*-value < 0.001) than those of fetal hypoglycemia. Detailed weight scores of the potential risks to the fetus and pregnancy are shown in Figure 3. Multiple comparisons are presented in Supplementary Table S1.

**Figure 4:**
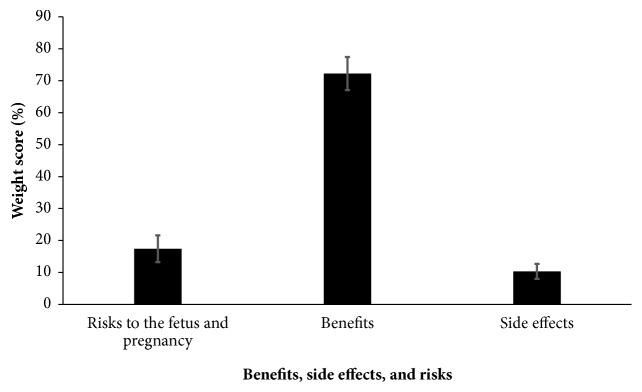
Weight scores of the benefits, side effects, and risks of ginger in the case presented.

When benefits were compared against the side effects and risks to the fetus and pregnancy, the former had significantly higher (*p*-value < 0.001) weight score (72.3%  ± 5.2%). Details of the weight scores are shown in Figure 4. Multiple comparisons are presented in Supplementary Table S1.

## 4. Discussion

The present study examined a case presentation and combined a professional and ethical deliberation method and a MCDA technique to weigh benefits against risks and facilitate a decision on using ginger for a pregnant woman suffering NVP along with other comorbidities. It is believed that healthcare providers use sophisticated forms of ethical and professional judgements in clinical practice to make decisions when faced with challenges in daily practice [56, 57]. Little research addressed how these hidden professional as well as ethical deliberations take place. This study aimed to analyze for the first time priorities of healthcare providers and patients when a medicinal plant offers potential benefits and at the same time has potential side effects and poses potential risks to the fetus and pregnancy, especially when multiple criteria contributed to the overall assessment. This study demonstrates that this combined method can be handy in facilitating shared decision making on using CAM modalities in certain cases. The study also demonstrates that the underpinnings of the decision making process were transparent and obvious to the stakeholders.

In this study, relevant benefits, side effects, and risks posed to the continuity of the pregnancy and the integrity of the developing fetus were weighed. On the grounds of these findings, light can be shed on the relative weights of these benefits, side effects, and risks in daily clinical practice. Using consensual benefits, in this study, arbitrary labeling and choice of benefits, side effects, and risks were avoided [58].

The AHP and the Utrecht methods were not used before either in combination or stand alone to facilitate decisions on CAM use. However, the AHP method was used in facilitating decision making in healthcare, regulatory affairs, and other decision making contexts [22, 59, 60].

Making decisions in CAM use can be complicated by a number of potential benefits, side effects, and other risks associated with the modality to be used, given the preferences of the two main stakeholders, the patient, and the healthcare provider. For example, in this study, the stakeholders gave relatively higher weight score for the potential of ginger in alleviating NVP in this case. As the woman was suffering from other comorbidities, high weight scores were also given to alleviating dyspepsia, flu, and cough. Other anticipated benefits of ginger were given comparatively lower weight scores. When the potential side effects were weighed, the stakeholders gave higher weight score to the potential risk of bleeding associated with ginger in this case. Similarly, the risks of heartburn, irritable bowel syndrome, and duodenal ulcer were given higher weight scores compared to other side effects. When the potential risks posed to the fetus and pregnancy were weighed, higher weight scores were given to the risk of spontaneous abortion and risks of impairing fetal development compared to inducing fetal hypoglycemia. When the potential benefits were weighed against the potential risks posed to the fetus and pregnancy and potential side effects, higher weight score was given to the potential benefits.

Probably, one of the major strengths of this study is the use of a combined method. While the AHP has previously proven handy in facilitating shared decision making [22, 59, 60], the Utrecht method can be handy in the deliberation process with a limited number of steps. This study can be a good example on facilitating deliberations in different contexts and thus can be easily translated to other cases and probably with other modalities of CAM.

It is important to mention that the findings apply to the particular case presented in this study; however, these findings are not meant to be generalizable to other cases or may not apply in another context. In other words, the ranking of benefits, side effects, and risks might be different for another case and might subsequently lead to different avenues in decision making. The goal of this study was to demonstrate that the Utrecht method and the AHP can be combined to facilitate professional and ethical decisions in CAM use. It is also noteworthy to mention that this combined method does not replace potential additional judgements, but simply furnishes a structured framework while explicitly considering the relative weights of each potential benefit, side effect, and risk associated with the use of a CAM modality and providing a clear starting point which subsequently facilitates deliberation and decision making.

In this study, the benefits, side effects, and risks were limited to those reported in the literature and on which formal consensus was achieved by a panel of gynecologists, physicians, and pregnant women [7]. Probably, there are other benefits, side effects, and risks that were not included in the deliberations and weighing process. This study is probably limited by the lack of data on many outcomes of using ginger for NVP. However, additional benefits, side effects, and risks could be included in the deliberation and weighing processes.

In this study, weighing the outcomes of using ginger in the presented case was done using the AHP. The AHP has a number of limitations that need to be considered while interpreting the findings [22, 61]. First, the AHP is known to be a time-consuming process. Second, structuring a dilemma with a large number of subcriteria creates more weights that need to be considered. Third, the scale 1-9 does not allow a 0 in case it applies. Fourth, the number of panelists was limited to 10. Although the number of panelists appear to be small, comparable sample size was used in previous studies [22, 62]. Moreover, the panel included different stakeholders who were physicians, pharmacists, herbalists, and pregnant women. Fifth, priorities might keep changing as a result of introducing or deleting alternatives. Finally, all alternatives are considered independent while in reality they might not be totally independent and some alternatives might be interrelated or dependent on one another.

## 5. Conclusion

In conclusion, this study demonstrates that the Utrecht method and the AHP can be combined and used to facilitate a shared decision in CAM practice.

### 5.1. How to Implement the Decision Preferred for This Case?

From the above results, clearly, we can see that ginger can be recommended for this woman as its potential benefits outweighed its potential side effects and risks to the fetus and pregnancy. In this case, the healthcare provider and the patient herself should take a shared decision to use ginger in her case. The woman should also be informed of the potential side effects and risks of using ginger. In this case, the use of Utrecht method and the AHP provided a framework for ethical as well as professional deliberation to decision making. The use of such methods promotes openness and transparency in decision making based on the perspectives of different stakeholders. Using these methods might help find common grounds between deliberators. It is important to take into consideration that no method of ethical and professional reflection can guarantee the best outcomes. Rather, the quality of outcomes depends on the quality of the ethical as well as professional deliberations themselves and the AHP depends on the perspectives of the stakeholders taken into consideration. Future studies are needed to evaluate the utility of this combined method to facilitate deliberations and decision making in CAM use. Using such methods might help promote transparency in making decisions in patient-centered care in daily CAM practice.

## Data Availability

Data supporting the results reported in this published article can be found in the Results section and as Supplementary Materials with this manuscript.

## Supplementary Materials

Supplementary Materials Supplementary Table S1 provides Bonferroni's multiple comparisons tests for subcriteria for benefits, side effects, and risk of ginger in the case presented.
