# Supplementary material for: Combining and Using the Utrecht Method and the Analytic Hierarchy Process to Facilitate Professional and Ethical Deliberation and Decision Making in Complementary and Alternative Medicine: A Case Study among a Panel of Stakeholders
Source: Evid Based Complement Alternat Med. 2018 Dec 23;2018:2315938. doi: 10.1155/2018/2315938 (PMC6323446; doi:10.1155/2018/2315938)
Supplement: Supplementary Materials — Supplementary Table S1 provides Bonferroni's multiple comparisons tests for subcriteria for benefits, side effects, and risk of ginger in the case presented. [file 2315938.f1.docx]

**Supplementary materials**

**Table S1:** Bonferroni's multiple comparisons tests for sub-criteria for benefits, side effects, and risk of ginger in the case presented

| **Bonferroni's multiple comparisons test** | | ***p*-value** |
| --- | --- | --- |
| **Benefits** |  |  |
| Alleviate NVP | Alleviate cough | 0.000 |
|  | Alleviate flu | 0.000 |
|  | Increase milk production | 0.000 |
|  | Decrease appetite | 0.000 |
|  | Decrease cholesterol levels | 0.000 |
|  | Reduce blood pressure | 0.000 |
|  | Reduce blood sugar levels | 0.000 |
|  | Alleviate dyspepsia | 0.000 |
|  | Improve sleep | 0.000 |
|  | Improve skin health | 0.000 |
|  | Reduce joint pain | 0.000 |
| Alleviate cough | Alleviate NVP | 0.000 |
|  | Alleviate flu | 1.000 |
|  | Increase milk production | 0.873 |
|  | Decrease appetite | 0.249 |
|  | Decrease cholesterol levels | 0.268 |
|  | Reduce blood pressure | 0.073 |
|  | Reduce blood sugar levels | 0.148 |
|  | Alleviate dyspepsia | 1.000 |
|  | Improve sleep | 0.382 |
|  | Improve skin health | 0.004 |
|  | Reduce joint pain | 0.013 |
| Alleviate flu | Alleviate NVP | 0.000 |
|  | Alleviate cough | 1.000 |
|  | Increase milk production | 1.000 |
|  | Decrease appetite | 0.447 |
|  | Decrease cholesterol levels | 0.478 |
|  | Reduce blood pressure | 0.138 |
|  | Reduce blood sugar levels | 0.271 |
|  | Alleviate dyspepsia | 0.890 |
|  | Improve sleep | 0.669 |
|  | Improve skin health | 0.009 |
|  | Reduce joint pain | 0.026 |
| Increase milk production | Alleviate NVP | 0.000 |
|  | Alleviate cough | 0.873 |
|  | Alleviate flu | 1.000 |
|  | Decrease appetite | 1.000 |
|  | Decrease cholesterol levels | 1.000 |
|  | Reduce blood pressure | 1.000 |
|  | Reduce blood sugar levels | 1.000 |
|  | Alleviate dyspepsia | 0.000 |
|  | Improve sleep | 1.000 |
|  | Improve skin health | 1.000 |
|  | Reduce joint pain | 1.000 |
| Decrease appetite | Alleviate NVP | 0.000 |
|  | Alleviate cough | 0.249 |
|  | Alleviate flu | 0.447 |
|  | Increase milk production | 1.000 |
|  | Decrease cholesterol levels | 1.000 |
|  | Reduce blood pressure | 1.000 |
|  | Reduce blood sugar levels | 1.000 |
|  | Alleviate dyspepsia | 0.000 |
|  | Improve sleep | 1.000 |
|  | Improve skin health | 1.000 |
|  | Reduce joint pain | 1.000 |
| Decrease cholesterol levels | Alleviate NVP | 0.000 |
|  | Alleviate cough | 0.268 |
|  | Alleviate flu | 0.478 |
|  | Increase milk production | 1.000 |
|  | Decrease appetite | 1.000 |
|  | Reduce blood pressure | 1.000 |
|  | Reduce blood sugar levels | 1.000 |
|  | Alleviate dyspepsia | 0.000 |
|  | Improve sleep | 1.000 |
|  | Improve skin health | 1.000 |
|  | Reduce joint pain | 1.000 |
| Reduce blood pressure | Alleviate NVP | 0.000 |
|  | Alleviate cough | 0.073 |
|  | Alleviate flu | 0.138 |
|  | Increase milk production | 1.000 |
|  | Decrease appetite | 1.000 |
|  | Decrease cholesterol levels | 1.000 |
|  | Reduce blood sugar levels | 1.000 |
|  | Alleviate dyspepsia | 0.000 |
|  | Improve sleep | 1.000 |
|  | Improve skin health | 1.000 |
|  | Reduce joint pain | 1.000 |
| Reduce blood sugar levels | Alleviate NVP | 0.000 |
|  | Alleviate cough | 0.148 |
|  | Alleviate flu | 0.271 |
|  | Increase milk production | 1.000 |
|  | Decrease appetite | 1.000 |
|  | Decrease cholesterol levels | 1.000 |
|  | Reduce blood pressure | 1.000 |
|  | Alleviate dyspepsia | 0.000 |
|  | Improve sleep | 1.000 |
|  | Improve skin health | 1.000 |
|  | Reduce joint pain | 1.000 |
| Alleviate dyspepsia | Alleviate NVP | 0.000 |
|  | Alleviate cough | 1.000 |
|  | Alleviate flu | 0.890 |
|  | Increase milk production | 0.000 |
|  | Decrease appetite | 0.000 |
|  | Decrease cholesterol levels | 0.000 |
|  | Reduce blood pressure | 0.000 |
|  | Reduce blood sugar levels | 0.000 |
|  | Improve sleep | 0.000 |
|  | Improve skin health | 0.000 |
|  | Reduce joint pain | 0.000 |
| Improve sleep | Alleviate NVP | 0.000 |
|  | Alleviate cough | 0.382 |
|  | Alleviate flu | 0.669 |
|  | Increase milk production | 1.000 |
|  | Decrease appetite | 1.000 |
|  | Decrease cholesterol levels | 1.000 |
|  | Reduce blood pressure | 1.000 |
|  | Reduce blood sugar levels | 1.000 |
|  | Alleviate dyspepsia | 0.000 |
|  | Improve skin health | 1.000 |
|  | Reduce joint pain | 1.000 |
| Improve skin health | Alleviate NVP | 0.000 |
|  | Alleviate cough | 0.004 |
|  | Alleviate flu | 0.009 |
|  | Increase milk production | 1.000 |
|  | Decrease appetite | 1.000 |
|  | Decrease cholesterol levels | 1.000 |
|  | Reduce blood pressure | 1.000 |
|  | Reduce blood sugar levels | 1.000 |
|  | Alleviate dyspepsia | 0.000 |
|  | Improve sleep | 1.000 |
|  | Reduce joint pain | 1.000 |
| Reduce joint pain | Alleviate NVP | 0.000 |
|  | Alleviate cough | 0.013 |
|  | Alleviate flu | 0.026 |
|  | Increase milk production | 1.000 |
|  | Decrease appetite | 1.000 |
|  | Decrease cholesterol levels | 1.000 |
|  | Reduce blood pressure | 1.000 |
|  | Reduce blood sugar levels | 1.000 |
|  | Alleviate dyspepsia | 0.000 |
|  | Improve sleep | 1.000 |
|  | Improve skin health | 1.000 |
| **Side effects** |  |  |
| Risk of bleeding | Cardiac arrhythmia | 0.000 |
|  | Irritable bowel syndrome | 0.000 |
|  | Duodenal ulcer | 0.000 |
|  | Heartburns | 0.000 |
|  | Hypotension | 0.000 |
|  | Hypoglycemia | 0.000 |
|  | Skin itching | 0.000 |
|  | Dehydration | 0.000 |
|  | Belching | 0.000 |
|  | Thirst | 0.000 |
|  | Sweating | 0.000 |
|  | Fever | 0.000 |
|  | Headache | 0.000 |
|  | Diarrhea | 0.000 |
| Cardiac arrhythmia | Risk of bleeding | 0.000 |
|  | Irritable bowel syndrome | 1.000 |
|  | Duodenal ulcer | 1.000 |
|  | Heartburns | 0.003 |
|  | Hypotension | 0.919 |
|  | Hypoglycemia | 1.000 |
|  | Skin itching | 1.000 |
|  | Dehydration | 1.000 |
|  | Belching | 1.000 |
|  | Thirst | 0.565 |
|  | Sweating | 0.585 |
|  | Fever | 0.936 |
|  | Headache | 0.569 |
|  | Diarrhea | 0.876 |
| Irritable bowel syndrome | Risk of bleeding | 0.000 |
|  | Cardiac arrhythmia | 1.000 |
|  | Duodenal ulcer | 1.000 |
|  | Heartburns | 1.000 |
|  | Hypotension | 0.002 |
|  | Hypoglycemia | 0.003 |
|  | Skin itching | 0.033 |
|  | Dehydration | 1.000 |
|  | Belching | 0.861 |
|  | Thirst | 0.001 |
|  | Sweating | 0.001 |
|  | Fever | 0.002 |
|  | Headache | 0.001 |
|  | Diarrhea | 0.002 |
| Duodenal ulcer | Risk of bleeding | 0.000 |
|  | Cardiac arrhythmia | 1.000 |
|  | Irritable bowel syndrome | 1.000 |
|  | Heartburns | 0.145 |
|  | Hypotension | 0.031 |
|  | Hypoglycemia | 0.044 |
|  | Skin itching | 0.354 |
|  | Dehydration | 1.000 |
|  | Belching | 1.000 |
|  | Thirst | 0.017 |
|  | Sweating | 0.018 |
|  | Fever | 0.032 |
|  | Headache | 0.017 |
|  | Diarrhea | 0.030 |
| Heartburns | Risk of bleeding | 0.000 |
|  | Cardiac arrhythmia | 0.003 |
|  | Irritable bowel syndrome | 1.000 |
|  | Duodenal ulcer | 0.145 |
|  | Hypotension | 0.000 |
|  | Hypoglycemia | 0.000 |
|  | Skin itching | 0.000 |
|  | Dehydration | 0.004 |
|  | Belching | 0.000 |
|  | Thirst | 0.000 |
|  | Sweating | 0.000 |
|  | Fever | 0.000 |
|  | Headache | 0.000 |
|  | Diarrhea | 0.000 |
| Hypotension | Risk of bleeding | 0.000 |
|  | Cardiac arrhythmia | 0.919 |
|  | Irritable bowel syndrome | 0.002 |
|  | Duodenal ulcer | 0.031 |
|  | Heartburns | 0.000 |
|  | Hypoglycemia | 1.000 |
|  | Skin itching | 1.000 |
|  | Dehydration | 0.714 |
|  | Belching | 1.000 |
|  | Thirst | 1.000 |
|  | Sweating | 1.000 |
|  | Fever | 1.000 |
|  | Headache | 1.000 |
|  | Diarrhea | 1.000 |
| Hypoglycemia | Risk of bleeding | 0.000 |
|  | Cardiac arrhythmia | 1.000 |
|  | Irritable bowel syndrome | 0.003 |
|  | Duodenal ulcer | 0.044 |
|  | Heartburns | 0.000 |
|  | Hypotension | 1.000 |
|  | Skin itching | 1.000 |
|  | Dehydration | 0.941 |
|  | Belching | 1.000 |
|  | Thirst | 1.000 |
|  | Sweating | 1.000 |
|  | Fever | 1.000 |
|  | Headache | 1.000 |
|  | Diarrhea | 1.000 |
| Skin itching | Risk of bleeding | 0.000 |
|  | Cardiac arrhythmia | 1.000 |
|  | Irritable bowel syndrome | 0.033 |
|  | Duodenal ulcer | 0.354 |
|  | Heartburns | 0.000 |
|  | Hypotension | 1.000 |
|  | Hypoglycemia | 1.000 |
|  | Dehydration | 1.000 |
|  | Belching | 1.000 |
|  | Thirst | 1.000 |
|  | Sweating | 1.000 |
|  | Fever | 1.000 |
|  | Headache | 1.000 |
|  | Diarrhea | 1.000 |
| Dehydration | Risk of bleeding | 0.000 |
|  | Cardiac arrhythmia | 1.000 |
|  | Irritable bowel syndrome | 1.000 |
|  | Duodenal ulcer | 1.000 |
|  | Heartburns | 0.004 |
|  | Hypotension | 0.714 |
|  | Hypoglycemia | 0.941 |
|  | Skin itching | 1.000 |
|  | Belching | 1.000 |
|  | Thirst | 0.434 |
|  | Sweating | 0.449 |
|  | Fever | 0.727 |
|  | Headache | 0.437 |
|  | Diarrhea | 0.679 |
| Belching | Risk of bleeding | 0.000 |
|  | Cardiac arrhythmia | 1.000 |
|  | Irritable bowel syndrome | 0.861 |
|  | Duodenal ulcer | 1.000 |
|  | Heartburns | 0.000 |
|  | Hypotension | 1.000 |
|  | Hypoglycemia | 1.000 |
|  | Skin itching | 1.000 |
|  | Dehydration | 1.000 |
|  | Thirst | 1.000 |
|  | Sweating | 1.000 |
|  | Fever | 1.000 |
|  | Headache | 1.000 |
|  | Diarrhea | 1.000 |
| Thirst | Risk of bleeding | 0.000 |
|  | Cardiac arrhythmia | 0.565 |
|  | Irritable bowel syndrome | 0.001 |
|  | Duodenal ulcer | 0.017 |
|  | Heartburns | 0.000 |
|  | Hypotension | 1.000 |
|  | Hypoglycemia | 1.000 |
|  | Skin itching | 1.000 |
|  | Dehydration | 0.434 |
|  | Belching | 1.000 |
|  | Sweating | 1.000 |
|  | Fever | 1.000 |
|  | Headache | 1.000 |
|  | Diarrhea | 1.000 |
| Sweating | Risk of bleeding | 0.000 |
|  | Cardiac arrhythmia | 0.585 |
|  | Irritable bowel syndrome | 0.001 |
|  | Duodenal ulcer | 0.018 |
|  | Heartburns | 0.000 |
|  | Hypotension | 1.000 |
|  | Hypoglycemia | 1.000 |
|  | Skin itching | 1.000 |
|  | Dehydration | 0.449 |
|  | Belching | 1.000 |
|  | Thirst | 1.000 |
|  | Fever | 1.000 |
|  | Headache | 1.000 |
|  | Diarrhea | 1.000 |
| Fever | Risk of bleeding | 0.000 |
|  | Cardiac arrhythmia | 0.936 |
|  | Irritable bowel syndrome | 0.002 |
|  | Duodenal ulcer | 0.032 |
|  | Heartburns | 0.000 |
|  | Hypotension | 1.000 |
|  | Hypoglycemia | 1.000 |
|  | Skin itching | 1.000 |
|  | Dehydration | 0.727 |
|  | Belching | 1.000 |
|  | Thirst | 1.000 |
|  | Sweating | 1.000 |
|  | Headache | 1.000 |
|  | Diarrhea | 1.000 |
| Headache | Risk of bleeding | 0.000 |
|  | Cardiac arrhythmia | 0.569 |
|  | Irritable bowel syndrome | 0.001 |
|  | Duodenal ulcer | 0.017 |
|  | Heartburns | 0.000 |
|  | Hypotension | 1.000 |
|  | Hypoglycemia | 1.000 |
|  | Skin itching | 1.000 |
|  | Dehydration | 0.437 |
|  | Belching | 1.000 |
|  | Thirst | 1.000 |
|  | Sweating | 1.000 |
|  | Fever | 1.000 |
|  | Diarrhea | 1.000 |
| Diarrhea | Risk of bleeding | 0.000 |
|  | Cardiac arrhythmia | 0.876 |
|  | Irritable bowel syndrome | 0.002 |
|  | Duodenal ulcer | 0.030 |
|  | Heartburns | 0.000 |
|  | Hypotension | 1.000 |
|  | Hypoglycemia | 1.000 |
|  | Skin itching | 1.000 |
|  | Dehydration | 0.679 |
|  | Belching | 1.000 |
|  | Thirst | 1.000 |
|  | Sweating | 1.000 |
|  | Fever | 1.000 |
|  | Headache | 1.000 |
| **Risks to the fetus and pregnancy** | |  |
| Risk of spontaneous abortion | Risk of impairment of fetal development | 0.144 |
|  | Risk of fetal hypoglycemia | 0.000 |
| Risk of impairment of fetal development | Risk of spontaneous abortion | 0.144 |
|  | Risk of fetal hypoglycemia | 0.000 |
| Risk of fetal hypoglycemia | Risk of spontaneous abortion | 0.000 |
|  | Risk of impairment of fetal development | 0.000 |
| **Benefits, side effects, and risks** | |  |
| Benefits | Side effects | 0.000 |
|  | Risks to the fetus and pregnancy | 0.000 |
| Side effects | Benefits | 0.000 |
|  | Risks to the fetus and pregnancy | 0.233 |
| Risks to the fetus and pregnancy | Benefits | 0.000 |
|  | Side effects | 0.233 |
